# Surface Modification of Medical-Grade Titanium and Polyvinyl Chloride with a Novel Catechol-Terminated Compound Containing Zwitterionic Sulfobetaine Functionality for Antibacterial Application

**DOI:** 10.3390/polym17152006

**Published:** 2025-07-22

**Authors:** Nai-Chia Fan, Fang-Min Hsu, Chi-Hui Cheng, Jui-Che Lin

**Affiliations:** 1 Division of Nephrology, Department of Pediatrics, Chang Gung Memorial Hospital, Taoyuan 333423, Taiwan; fannaichia@gmail.com (N.-C.F.); pedneph.cheng@msa.hinet.net (C.-H.C.); 2 Graduate Institute of Clinical Medical Sciences, Chang Gung University, Taoyuan 333323, Taiwan; 3 Department of Chemical Engineering, National Cheng Kung University, Tainan 701401, Taiwan; fangminsyu@gmail.com; 4 Department of Pediatrics, College of Medicine, Chang Gung University, Taoyuan 333323, Taiwan; 5 School of Dentistry, Institute of Oral Medicine, College of Medicine, National Cheng Kung University, Tainan 701401, Taiwan

**Keywords:** zwitterionic, dopamine, antibacterial, mussel-inspired, surface modification

## Abstract

Healthcare-associated infection, mainly through medical device-associated infection, remains a critical issue in hospital care. Bacterial adhesion, proliferation, and biofilm formation on the device surface have been considered the foremost cause of medical device-associated infection. Different means have been explored to reduce microbial attachment and proliferation, including forming a bactericidal or microbial adhesion-resistant surface layer. Fear of limited bactericidal capability if the dead microbes remained adhered to the surface has withheld the widespread use of a bactericidal surface in medical devices if it was intended for long-term use. By contrast, constructing a microbial adhesion-resistant or antifouling surface, such as a surface with zwitterionic functionality, would be more feasible for devices intended to be used for the long term. Nevertheless, a sophisticated multi-step chemical reaction process would be needed. Instead, a simple immersion method that utilized a novel mussel-inspired catechol compound with zwitterionic sulfobetaine functionality, ZDS, was explored in this investigation for the surface modification of substrates with distinctively different surface characteristics, including titanium and polyvinyl chloride. Dopamine, NaIO_4_ oxidants, and chemicals that could affect ionic interactions (NaCl and polyethyleneimine) were added to the ZDS-containing immersion solution to compare their effects on modifying titanium and PVC substrates. Furthermore, a layer-by-layer immersion method, in which the substrate was first immersed in the no-ZDS-added dopamine-containing solution, followed by the ZDS-containing solution, was also attempted on the PVC substrate. By properly selecting the immersion solution formulation and additional NaIO_4_ oxidation modification, the antibacterial capability of ZDS-modified substrates can be optimized without causing cytotoxicity. The maximum antibacterial percentages against *S. aureus* were 84.2% and 81.7% for the modified titanium and PVC substrate, respectively, and both modified surfaces did not show any cytotoxicity.

## 1. Introduction

Healthcare-associated infections (HAIs) remain an issue of focus in basic and clinical medicine research. HAI refers to patients infected with microbes during their hospital stay or while undergoing medical treatments [1,2,3]. Most HAIs could be attributed to the microbial attachment to the surface of medical devices. Once the biofilms are formed following bacterial adhesion to the device surfaces, more aggressive medical treatment would be required to avoid severe complications and long-term sequels.

To reduce HAIs, various approaches have been explored to reduce microbial adhesion and/or kill the microbes once adhered or before adhesion to the surface [4,5,6]. Nevertheless, the emergence of antibiotic-resistant/biocide-resistant strains has been of concern if the antibiotic or biocide is used as the controlled-release agent from the medical device. On the other hand, creating a bactericidal surface, such as the surface functionalized with cationic groups, for example, pyridinium, phosphonium, and quaternary ammonium functionalities, could obviate the issue of antibiotic-resistant strains [3,7,8,9]. Nonetheless, concern about the dead bacteria remaining adhered to the surfaces can limit their further bactericidal effect through the contact-killing mechanism [2,4,10].

Reducing or even preventing bacterial adhesion onto the medical device surface would be another alternative to mitigate the problems associated with HAIs. Polyethylene glycol has been considered an effective compound for reducing microbial attachment, protein adsorption, and even biofouling on various biomedical devices. This can be attributed to the hydration layer formed by the hydrogen bonding and the repulsion force against the subsequently approaching biomolecules, so-called the excluded volume effect [11,12,13,14,15]. Nevertheless, the likely oxidation by the air, heat, and enzyme or metal ions in the physiological environment has limited the PEG use for long-term application [16,17]. Compounds terminated with zwitterionic functionality, including sulfobetaine, carboxybetaine, phosphobetaine, and biomimetic phosphorylcholine chemical configurations, have been shown to reduce protein adsorption, reduce microbial adhesion, reduce cellular attachment, and improve the materials’ antifouling property and biocompatibility. This is attributed to the hydration layer formed due to the electrostatic interactions that lead to the antifouling characteristics [18,19,20,21,22,23].

A mussel-inspired surface modification technique that utilizes dopamine or catechol-based derivatives has received significant attention lately due to the mussel’s capability to bind to a wide variety of organic and inorganic substrates [24,25,26,27,28,29,30,31]. The mechanistic details governing the mussel-inspired deposition/surface modification process for different substrates remain elusive. The type of substrate and deposition solution formulation could affect the interactions among the substrate and solutes through various molecular interactions, such as π–π stacking, cation–π interactions, hydrogen bonding, covalent bonding, and hydrophobic–hydrophobic interactions, that eventually lead to different surface characteristics and bio-contacting properties of the deposit/surface layer [32,33,34,35].

Despite such complicated mechanisms, different processes were explored to functionalize the surface with zwitterionic groups using the mussel-inspired surface deposition/grafting pathways. Direct co-deposition of dopamine and zwitterionic monomer, such as sulfobetaine methacrylate (SBMA), has been attempted to create a surface layer immobilized with 5,6-indolequinone-terminated zwitterionic polymer [36]. On the other hand, copolymers prepared by a monomer with a dopamine-like configuration, dopamine methacrylamide (DMA), and zwitterionic monomers were also reported for direct deposition onto the bare substrate [37]. Co-deposition of dopamine and zwitterionic polymer, such as poly(sulfobetaine methacrylate) (PSBMA), was also reported on different substrates [38]. Instead of direct deposition onto the non-modified substrate mentioned above, studies have utilized sequential modification steps by first modifying the substrates with dopamine or dopamine and polyethylenimine (PEI), then with the zwitterionic polymers through the amino-groups-mediated surface grafting reaction scheme [38,39,40]. As opposed to the zwitterionic polymers, a zwitterionic dopamine derivative (ZW-DOPA) was synthesized and used for the surface modification of different substrates, and the surface characteristics of the modified layer were optimized by selecting the proper oxidizing conditions, including the pH value of the coating solution and oxidizing agents used [41].

Rather than the complicated synthesis steps to prepare the ZW-DOPA, as reported by Yeon et al. [41], a simpler procedure was explored in this study to synthesize a novel compound, ZDS (3-[2-(3,4-dihydroxyphenyl)ethylaminium]propane-1-sulfonate), containing the dopamine-like terminal end and zwitterionic sulfobetaine functionality. Different surface modification/coating schemes were attempted to change the surface characteristics and to optimize the antimicrobial properties of medical-grade titanium and polyvinyl chloride (PVC). Various additives, including dopamine, and those that could influence the ionic interactions within the immersion/coating solution, such as PEI and NaCl, were added to the ZDS-containing immersion solution. Addition of the oxidant, NaIO_4_, was also explored, since the oxidants have been indicated to increase the polydopamine deposition rate and to enhance the conversion of catechol to a quinone structure [41,42,43]. Changing the ionic strength of the immersion buffer was also attempted. Besides the one-layer approach utilizing different ZDS-containing deposition solutions in the first treatment step, a layer-by-layer immersion method, in which the substrate was first immersed by the no-ZDS-added dopamine-containing solution, then followed by the ZDS solution, was also attempted on the PVC substrate. The different modified specimens’ surface characteristics, antimicrobial capability, and cytotoxicity were investigated and compared.

## 2. Materials and Methods

### 2.1. Materials

Chemicals and solvents used for the synthesis and surface modification, including dopamine hydrochloride (DA), 1,3-propane sultone, iodomethane, dimethylformamide (DMF), diethyl ether, ammonium hydroxide, ethanol (EtOH), sodium carbonate, tris(hydroxymethyl)aminomethane (Trizma^®^ base), sulfuric acid, hydrogen peroxide, sodium periodate (NaIO_4_), sodium chloride (NaCl), and polyethyleneimine (PEI, MW: 600), were purchased from different vendors, including Sigma-Aldrich (St. Louis, MO, USA), Fluka(Charlotte, NC, USA), Alfa Aesar (Ward Hill, MA, USA), J.T. Baker (Avantor, Radnor, PA, USA), Thermo Fisher Scientific (Waltham, MA, USA), and MACRON (Avantor, Radnor, PA, USA), at the highest purity available through two different Taiwanese vendors. The substrates used for surface modification were titanium (Ti, grade I cp, thickness 0.2 cm, AcrUshin Co., Chiyoda-ku, Tokyo, Japan) and polyvinyl chloride sheet (PVC, Nan Ya Plastics Corp, Taiwan).

For the antibacterial assay and cytotoxicity assessment, soya peptone, tryptone type-1, agar, sodium chloride, sodium hydroxide, ethanol, Minimum Essential Medium (MEM), Horse serum, Penicillin-Streptomycin (P/S (100×)), GlutaMAXTM-1 (100×), HEPES solution (1 M, pH 7.0–7.6), MEM Non-Essential Amino Acid Solution (100×), Sodium Pyruvate solution, phosphate-buffered saline (PBS, pH 7.2), isopropanol (>99.5% purity), dimethyl sulfoxide (DMSO, >99.5% purity), and 3-(4,5-dimethylthiazol-2-yl)-2,5-diphenyltetrazolium bromide (MTT) were acquired from HIMEDIA (Mumbai, Maharashtra, India), Becton Dickinson (Franklin Lakes, NJ, USA), Sigma-Aldrich, Honeywell International (Charlotte, NC, USA), J.T. Baker, Gibco (Waltham, MA, USA), and Thermo Fisher Scientific at the highest grade/purity available. The microbial strains, namely *S. aureus* (ATCC 21351) and L929 mouse fibroblast cells (NCTC clone 929, BCRC-RM60091), were acquired from the Food Industry Research and Development Institute in Hsinchu, Taiwan.

### 2.2. Synthesis of ZDS

**Scheme 1:**
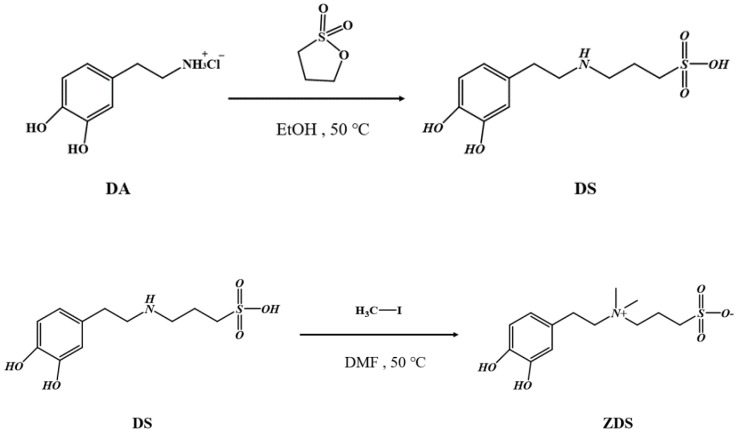
The synthesis scheme for ZDS.

The ZDS, 3-[2-(3,4-dihydroxyphenyl)ethylaminium]propane-1-sulfonate, synthesis scheme was based on Wei et al. [44] and modified to achieve the desired purity and yield (Scheme 1).

First, 1.9 g of DA was added to a two-necked flask containing 150 mL of EtOH. A condenser was then connected to the flask. The system was then vacuumed and filled with argon. Subsequently, 1.35 g of 1,3-propane sultone and 690 μL of ammonium hydroxide were added by syringe injection for reaction at 50 °C for three days. After the reaction, the solution was centrifuged at 6000 rpm for 25 min at room temperature to remove the EtOH liquid waste. Then, the clean EtOH was added to disperse/wash the crude product, which was repeated three times. The final solid white product, DS (3-[2-(3,4-dihydroxyphenyl)ethylamino]propane-1-sulfonic acid), was then dried in the oven with a yield of 80%.

A total of 0.33 g of DS was added to a double-necked flask containing 150 mL of DMF. Then, 0.25 g of sodium carbonate was added to the flask above. The flask was then vacuumed, filled with argon, and kept in an ice bath for 30 min. In the ice bath, 2.2 mL of iodomethane was then injected and stirred for 10 min. The ice bath was removed, and the reaction was executed at 50 °C for 20 h. After the reaction, the solvent was removed by Rotavapor. The crude product was then cold-crystallized and precipitated in the refrigerator overnight after adding 60 mL of diethyl ether. The diethyl ether was then removed by simple filtration. The crude product on filter paper was rinsed twice with a copious amount of deionized water. The precipitate was then freeze-dried to remove the residual water. The final solid white product, ZDS (3-[2-(3,4 dihydroxyphenyl)ethylaminium]propane-1-sulfonate), was obtained with a yield of 95%.

### 2.3. Surface Modification of Different Substrates

#### 2.3.1. Titanium (Ti) Substrate

The titanium substrate (1 cm × 1 cm) was cleaned with neutral detergent, deionized water, ethanol, and acetone sequentially for 15 min each in the ultrasonicator. The cleaned titanium substrate was purge-dried with argon. The dried titanium substrate was further immersed in the piranha solution for 1 h. The substrate was then washed with deionized water ultrasonically for 5 min, blow-dried with argon, and then stored in the methanol solution before further surface modification.

The coating solution was prepared with 2 mg/mL of ZDS in a Tris buffer (10 mM, pH = 8.50). The piranha-modified titanium substrate was immersed in the coating solution for 24 h at 30 °C. Different concentrations of dopamine (DA) or 10 mM of the sodium chloride solution were also added to the ZDS coating solution to examine the effect of the solution formulation on the coating quality.

Following the 24 h of immersion, some coated titanium substrates were further immersed in the oxidant, 50 mM of the NaIO_4_ aqueous solution, for 30 min. By comparison, a lower concentration of the NaIO_4_ aqueous solution, 20 mM, was added into the 2 mg/mL of the ZDS coating solution for a shorter coating duration of 2 h at 30 °C due to a fast deposition rate [42,43], as shown in the results section.

All modified titanium substrates were ultrasonically cleaned in deionized water and then blow-dried with argon before surface analyses and bioassays. The volume of the different immersion solutions was kept at 20 mL. At least three samples were prepared for different modified groups.

The nomenclature and the parameters used for the surface modification are shown in Table 1 and Table S1.

#### 2.3.2. Polyvinyl Chloride (PVC) Substrate

The polyvinyl chloride sheet was cut into pieces of 1 cm × 1 cm in size and then cleaned with a neutral detergent, deionized water, and ethanol in sequence for 15 min each in an ultrasonicator. The cleaned PVC substrate was then stored in a vacuum oven before further use.

Similar to the surface modification for the Ti substrate, the coating solution was prepared with 2 mg/mL of ZDS in a Tris buffer (10 mM, pH = 8.50). Different concentrations of dopamine (DA), 10 mM of the sodium chloride solution, and 1 mg/mL of PEI (MW: 600) were also added to the ZDS coating solution to explore their effects on surface modification. For the coating solution containing PEI, an additional Tris buffer at higher molarity (50 mM, pH = 8.50) was used to examine the ionic strength effect on the surface coating process. The PVC substrate was immersed in the coating solution for 24 h at 30 °C.

Similar to the surface modification scheme for the Ti substrates, some modified PVC substrates were further immersed in the oxidant, 50 mM of the NaIO_4_ aqueous solution, for 30 min. A lower amount of 20 mM of the NaIO_4_ aqueous solution was added into the 2 mg/mL of the ZDS coating solution for a shorter coating duration of 2 h at 30 °C to examine the likely effects of oxidants in the surface modification/deposition for PVC substrates.

All modified PVC substrates were then ultrasonically cleaned in deionized water to remove the physically bound or loosely bound substances, then blow-dried with argon, and stored in a vacuum oven before surface analyses and bioassays. The solution volume of each immersion step was 20 mL, the same as that for the titanium modification. At least three samples were prepared for each modification group.

The nomenclature and the parameters used for the surface modification of PVC are shown in Table 2 and Table S2.

Besides the one-layer approach mentioned above, a “layer-by-layer” surface modification scheme was utilized to modify the PVC substrate, in which the DA-containing solution was used to build the first layer, followed by immersion in the ZDS-containing solution for the second deposit layer (Table 3 and Table S3). To construct the first layer of the surface deposit, the cleaned PVC substrate was immersed in 2 mg/mL of the DA Tris buffer (10 mM, pH = 8.50) with or without PEI (1 mg/mL) at 30 °C for 24 h. Using the 2 mg/mL of DA as the 2 mg/mL of ZDS in the previous one-layer approach was intended to explore the effects associated with the chemical configuration between these two compounds. The effect of using a Tris buffer at higher molarity, 50 mM at pH = 8.50, was also explored.

Following the deionized water rinsing, the 1st-layer of the modified PVC substrate was immersed in the 5 mg/mL of ZDS Tris buffer (10 mM or 50 mM, pH = 8.50) with or without PEI (2.5 mg/mL) at 30 °C for 24 h. The higher ZDS concentration used, 5 mg/L, as compared to 2 mg/mL of ZDS used in the earlier one-layer approach, was to ensure the surface hydrophilicity in the 2nd layer deposit (see contact angle analysis for the one-layer and the layer-by-layer approach for the PVC surface modification). The ZDS/PEI ratio (2/1) was the same as the DA/PEI ratio in the first-step immersion solution. After being immersed in the second-layer solution, the PVC samples were ultrasonically cleaned in deionized water to remove the physically bound or loosely bound substances, blow-dried with argon, and stored in a vacuum oven before surface analyses and bioassays. The volume of each immersion step and the number of samples prepared for each group were the same as those used for the one-layer approach.

The nomenclature and the parameters used for the layer-by-layer surface modification of PVC are shown in Table 3 and Table S3.

### 2.4. Characterization

The chemical structure of ZDS was verified by nuclear magnetic resonance (NMR) (Bruker AV-500, Zurich, Switzerland). Different surface analysis methods were employed to evaluate the various surface properties of the modified substrate fully. The surface hydrophobicity, surface morphology, and chemical bonding state and element composition of the modified surface were analyzed by water contact angle (WCA) (Model 100SB, Sindatek Instrument Co., Ltd., Taiwan), scanning electron microscope (SEM) (SU8010, Hitachi, Tokyo, Japan), and X-ray photoelectron spectroscopy (XPS) (PHI Quantera II, ULVAC-PHI, Kanagawa, Japan), respectively.

### 2.5. Antibacterial Test

The nascent or modified substrates were placed into a 24-well plate. Subsequently, 2 mL of bacteria suspension (2 × 10^6^ CFU/mL) was introduced into each well and incubated for 3 h at 37 °C. After incubation, the samples were gently washed with PBS three times on both the front and rear sides to remove non-adhered bacteria. The washed samples were then ultrasonicated at 200 W, 40 KHz for 5 min to detach the adhered microbes. Finally, the detached microbial suspension was appropriately diluted and spread onto the agar plates to count the colonies and to determine microbial viability. The antibacterial activity was determined by the bacterial reduction percentage compared to the non-modified control substrate.

### 2.6. Cytotoxicity Assay

A cytotoxicity test was conducted based on the standard protocol of ISO 10993-5 [45] and ISO 10993-12 [46] using the L929 mouse fibroblast cells (NCTC clone 929, BCRC-RM60091) by the extraction method [3]. The L929 cell suspension was cultured in the Minimum Essential Medium (MEM) medium, containing 10% Horse serum (HS), 1% Penicillin-Streptomycin (P/S), 1% HEPES solution, 1% MEM Non-essential Amino Acid Solution (100×), 1% Sodium Pyruvate solution, 1% GlutaMAXTM-1 (100×) at 37 °C, and 5% CO_2_, and the medium was replaced every three days.

The samples were sterilized by soaking in 75% ethanol at 4 °C for 15 min and then rinsed 3 times with a sterilized potassium phosphate buffer solution (PBS). The sample eluent was prepared by immersing the sterilized sample in 0.65 mL of medium for 24 h; additionally, the medium without a sample served as the control.

The L929 cells were seeded in a 96-well plate with a density of 1 × 10^5^ cells/well, and incubated in 5% CO_2_ at 37 °C for 24 h. The medium was subsequently replaced with the sample eluent. Following an additional 24 h of culture at 37 °C and 5% CO_2_ atmosphere, the cell viability was examined using the MTT assay by determining the absorbance at 570 nm using the Enzyme-Linked Immunosorbent Assay (ELISA). The reference wavelength was set at 650 nm. Polyethylene plastic wrap and latex gloves were used as the negative and positive controls for the cytotoxicity testing. The culture medium served as the control. Three samples were analyzed in each category, and the cell viability was normalized to the control.

## 3. Results and Discussion

### 3.1. ZDS Synthesis

**Figure 1:**
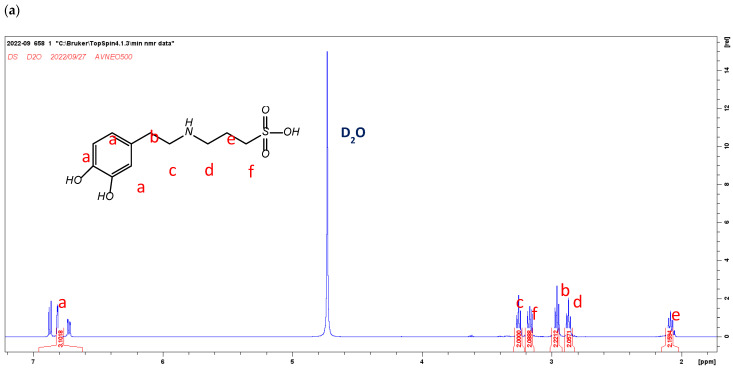

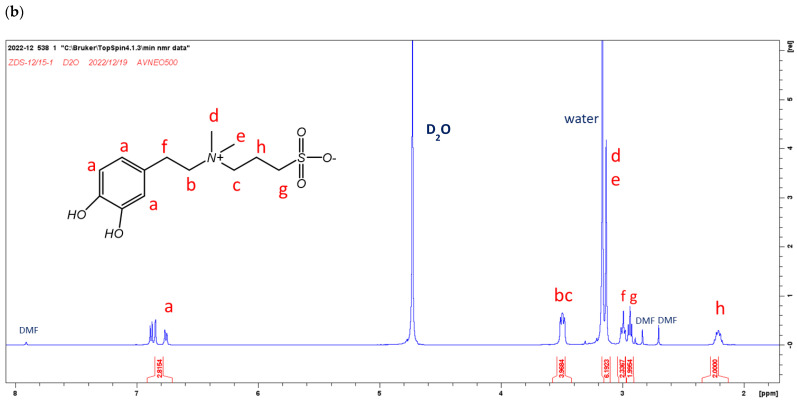
The ^1^H-NMR spectra of (**a**) DS (400 MHz, D_2_O): δ (ppm): 2.08 (m, 2H), 2.88–2.90 (m, 2H), 2.94–2.99 (m, 2H), 3.14–3.17 (m, 2H), 3.26 (m, 2H), 6.71–6.74 (m, 1H), 6.82–6.88 (m, 2H); and (**b**) ZDS (400 MHz, D_2_O) δ (ppm): 2.12 (m, 2H), 2.85 (t, 2H), 2.91 (m, 2H), 3.05 (s, 6H), 3.41 (m,4H), 6.67 (m, 1H), 6.76 (d, 1H), 6.80 (d, 1H).

The ^1^H-NMR spectra of DS and ZDS are shown in Figure 1a,b. The NMR peak assignment of DS and ZDS was referred from the studies by Wei et al. [44] and Ferretti et al. [47], respectively. The NMR spectra indicated that all these compounds were well-prepared and of reasonably high purity.

### 3.2. Surface Characterization

#### 3.2.1. Surface Morphology

##### Surface Morphology of the Modified Titanium Substrates

**Figure 2:**
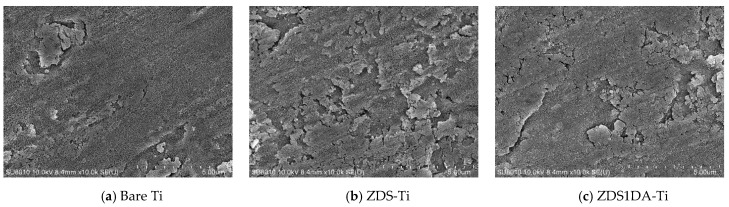

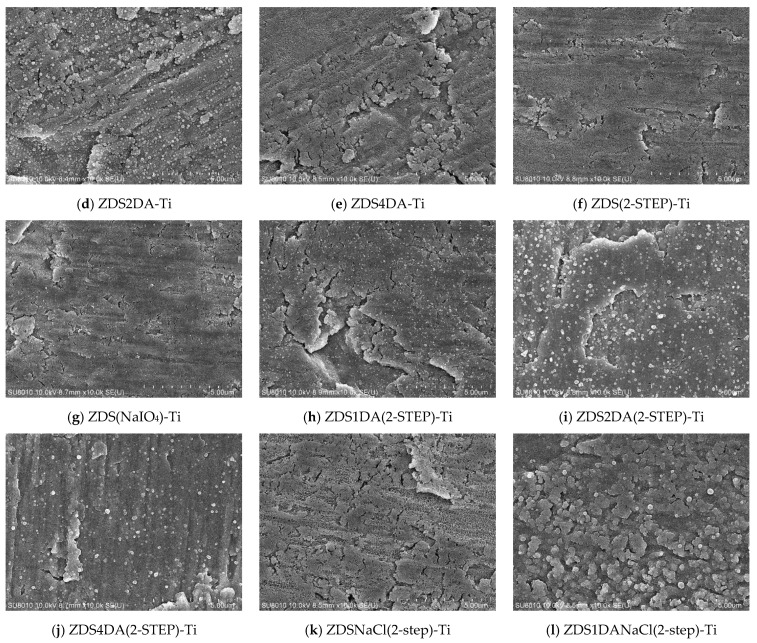
The SEM micrographs of different Ti substrates (×10 k).

The surface morphology of the bare titanium and different modified titanium substrates is shown in Figure 2. Likely resulting from the piranha cleaning/etching effect, the bare titanium presented few pits and a coral-like structure (Figure 2a). The titanium substrates modified with the dopamine (DA)-added solution generally presented a thicker aggregate/deposited layer than those without counterparts. Further, the surface roughness/aggregates appeared to increase with the amount of DA added (Figure 2c–e,h–j). This could be attributed to the self-polymerization/aggregation of DA and the copolymerization/aggregation of DA and ZDS.

The additional NaIO_4_ oxidant treatment step (sample nomenclature with (2-STEP), e.g., ZDS1DA(2-STEP)-Ti (Figure 2h)) appears to increase the surface roughness with the notion of particulate aggregates. The NaIO_4_ treatment also increased the deposit thickness when ZDS was the only compound in the coating solution, no matter whether it was directly added into the coating solution (ZDS(NaIO_4_)-Ti (Figure 2g)) or used as an additional deposition solution (ZDS(2-STEP)-Ti (Figure 2f)). These findings could be due to the enhanced polymerization/aggregation of DA/DA, ZDS/ZDS, and DA/ZDS due to the NaIO_4_ oxidation effect [42,43]; even a shorter 2-h or 30-min immersion step was utilized.

The addition of 10 mM NaCl in the coating solution appeared to increase the surface roughness with the notion of small holes and a coral-like structure (i.e., ZDSNaCl(2-STEP)-Ti (Figure 2k) vs. ZDS(2-STEP)-Ti (Figure 2f)). This may be due to the electrostatic interactions between the salt ions and the zwitterionic terminal ends of ZDS [48], leading to easier access for the surface-adsorbed ZDS to undergo self-polymerization/aggregation by the subsequent NaIO_4_ oxidation. This explanation was further substantiated when a rougher surface was noted with the DA addition (i.e., ZDS1DANaCl(2-STEP)-Ti (Figure 2l) vs. ZDS1DA(2-STEP)-Ti (Figure 2h)).

##### Surface Morphology of the Modified Polyvinyl Chloride Substrates by the One-Layer Approach

**Figure 3:**
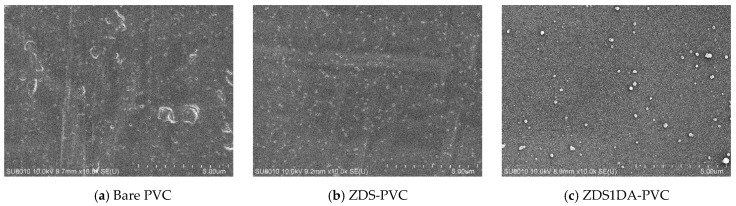

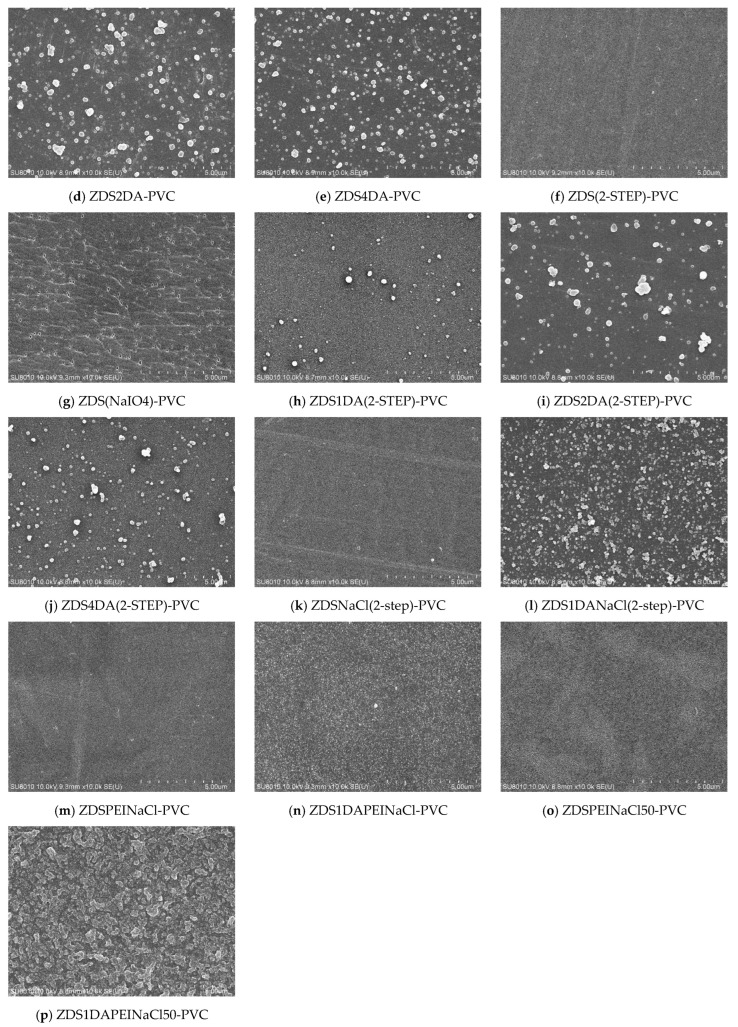
The SEM micrographs of different PVC substrates modified by the one-layer approach (×10 k).

Likely resulting from the mechanical rolling and blowing steps during the film preparation, the bare, non-treated PVC exhibited scratches and holes even after multiple cleaning steps (Figure 3a). Similar to the Ti substrates, adding DA to the ZDS-based coating solution would increase surface roughness and particulate deposition with the amount of DA added (Figure 3c–e,h–j). By contrast, the PVC modified with the pure ZDS solution exhibited a smoother surface than the bare PVC, although tiny holes were noted (Figure 3b). Nevertheless, these small holes almost disappeared if an additional NaIO_4_ oxidant immersion step was followed, even for only a 30-min immersion (ZDS(2-STEP)-PVC) (Figure 3f). This could be attributed to the enhanced oxidation/polymerization of the surface-bound ZDS with NaIO_4_ [42,43]. By contrast, if the oxidant NaIO_4_ was added to the ZDS coating solution, the ZDS(NaIO_4_)-PVC substrate exhibited fibril streaks and tiny particles on a rough surface (Figure 3g). This could be attributed to the enhanced oxidation/polymerization of ZDS in the solution phase, not in the substrate as ZDS(2-STEP)-PVC mentioned above (Figure 3f), which led to the tiny particles/fibrils adsorption/binding onto the PVC substrate. In contrast to the Ti substrate, the addition of 10 mM NaCl in the coating solution did not increase the modified PVC surface roughness (ZDSNaCl(2-STEP)-PVC (Figure 3k) vs. ZDS(2-STEP)-PVC (Figure 3f)). We speculate this may be due to the differences in the interactions between ZDS and the substrate (PVC vs. Ti). Nevertheless, adding the DA into the ZDS + NaCl coating solution still led to an increase in the surface roughness of the modified PVC substrate (ZDS1DANaCl(2-STEP)-PVC (Figure 3l) vs. ZDSNaCl(2-STEP)PVC (Figure 3k)) as Ti one.

To explore the likely effects caused by the charged long alkyl chains on the deposition of the zwitterionic ZDS on the hydrophobic PVC substrate, 1 mg/mL polyethyleneimine (PEI, MW: 600) bearing primary and secondary amines was added to the coating solution containing ZDS and 10 mM NaCl. Meanwhile, to strengthen the pH buffering effect after adding the charged PEI into the deposition solution, a 50 mM Tris buffer at pH = 8.50 was utilized, in addition to the 10 mM Tris buffer at pH = 8.50 for preparing the deposition/coating solution. Using the 10 mM Tris buffer, the ZDSPEINaCl-PVC (Figure 3m) appeared smoother than the ZDS-PVC (Figure 3b). This is in line with another study in which PEI could suppress the precipitation of solution aggregates, leading to a more uniform coating [49]. Nevertheless, further adding the DA to the solution led to a surface with various small particulate deposits (ZDS1DAPEINaCl-PVC (Figure 3n) vs. ZDSPEINaCl-PVC (Figure 3m)). The surfaces modified by the Tris buffer with higher ionic strength (i.e., 50 mM) exhibited increases in surface particulate formation and roughness, compared to the counterparts modified by the Tris buffer at 10 mM (e.g., ZDS1DAPEINaCl50-PVC (Figure 3p) vs. ZDS1DAPEINaCl-PVC (Figure 3n)). All these findings suggested that tuning the ionic interactions among the charged solutes in the deposition solution is a crucial step in controlling the deposit quality.

##### Surface Morphology of the Modified Polyvinyl Chloride Substrates by the Layer-by-Layer Approach

**Figure 4:**
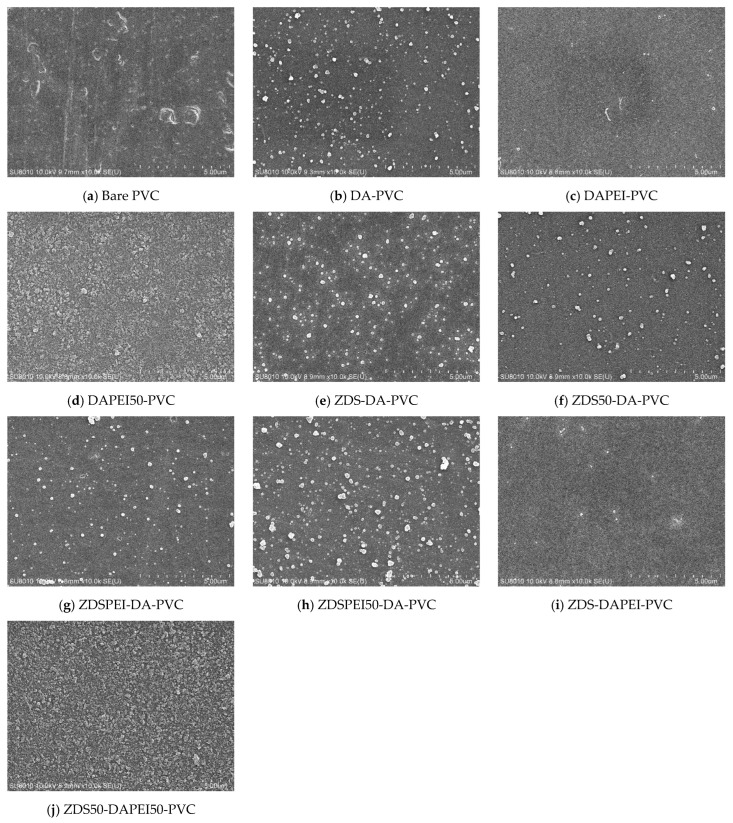
The SEM micrographs of PVC substrates modified by layer-by-layer methods (×10 k).

For the layer-by-layer approach, the first layer prepared by immersing the PVC substrate into the DA-containing solution presented a rough surface with a micro-particulate deposit (Figure 4b–d), similar to what was shown in the one-layer approach using the DA-containing solution (Figure 3c–e,h–j,l,n,p). Depending on the additive and buffer molarity (i.e., PEI and 50 mM Tris buffer), this first layer presented with different sizes of particulates and roughness. For the second layer prepared with the ZDS-containing buffer, various surface features were noted compared to the first layer, except for those prepared with only ZDS in the second immersion solution (Figure 4e,i). The close resemblance of surface features of ZDS-DA-PVC (Figure 4e) vs. DA-PVC (Figure 4b) and ZDS-DAPEI-PVC (Figure 4i) vs. DAPEI-PVC (Figure 4c) implies that the second layer of the ZDS deposit may be too thin to change the surface features of the first layer. As expected, adding the PEI or changing the Tris buffer molarity in the ZDS coating solution would change the interactions among the solution molecules and the surface-bound molecules in the previously formed first layer, leading to a distinct second-layer morphology. Among these layer-by-layer PVC modified substrates, the ZDS50-DAPEI50-PVC (Figure 4j) presented a distinctive deposit with the uniform coating of tiny particulates, which was rarely noted in others.

#### 3.2.2. Surface Contact Angle

##### Surface Contact Angle of the Modified Titanium Substrates

**Figure 5:**
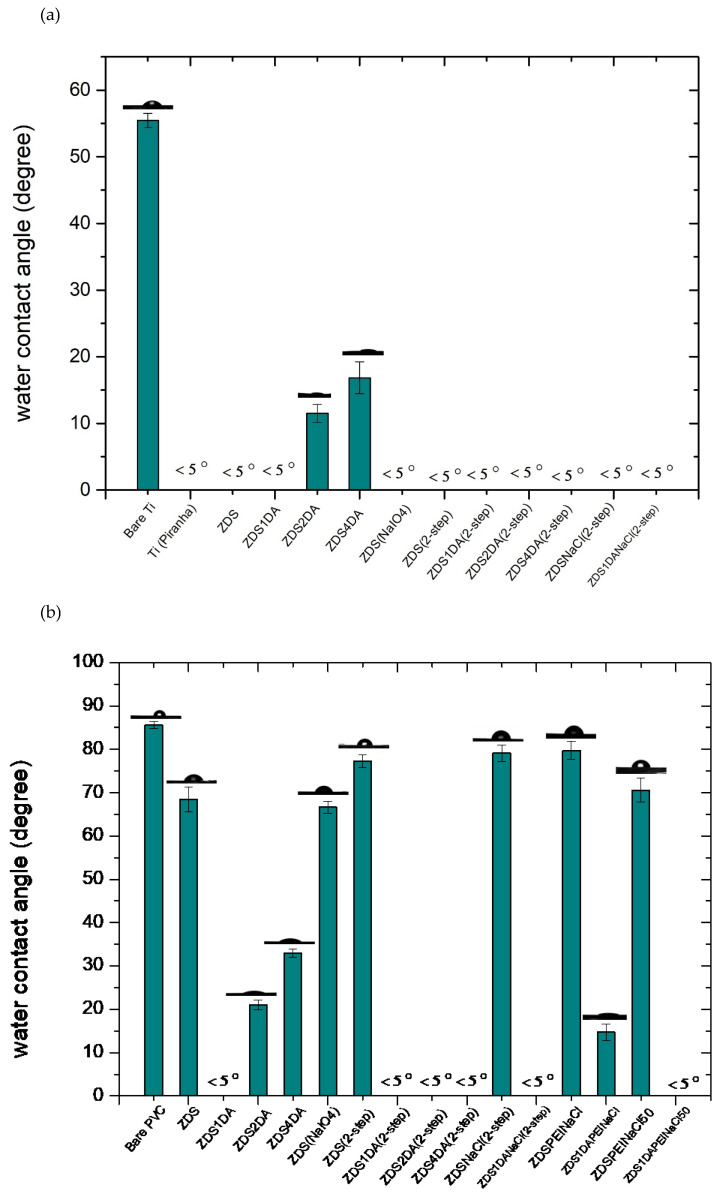
The static water contact angle value of different (**a**) titanium and (**b**) PVC substrates by the one-layer approach.

For the titanium substrate, the water contact angle of the cleaned titanium substrate (Bare Ti) is about 55°, while it is reduced to <5° after being treated with the piranha solution (Ti piranha; Figure 5a), likely due to the formation of titanol (Ti-OH) after piranha oxidation. The higher contact angle value noted in the cleaned titanium substrate (Bare Ti) could be due to the surface adsorbed adventitious hydrocarbons (see Section 3.2.3) or the surface titanoxane (Ti-O-Ti) formed after being exposed to an oxygen-containing environment or irradiated with visible light [50].

Most of the titanium modified surfaces exhibited very hydrophilic characteristics (<5°) except for those modified with the ZDS + 2DA and ZDS + 4DA solutions, in which the contact angle values were 11.50 ± 1.36° and 16.84 ± 2.39°, respectively. The zwitterionic functionalities associated with ZDS could lead to a very low water contact angle on the modified titanium surface. Therefore, such an increase in water contact angle with the amount of DA added could be attributed to the more hydrophobic backbone and/or thicker deposit formed with the addition of DA. The use of the NaIO_4_ oxidant, either directly added into the coating solution or used as the second oxidation step for the layer formed, will render the surface very hydrophilic, even if the previous layers formed were hydrophobic, namely the ZDS + 2DA and ZDS + 4DA modified Ti, or the addition of 10 mM NaCl that was intended to change/shield the electrostatic interactions between the positive and negative charges (i.e., zwitterionic functionalities) of ZDS [48].

##### Surface Contact Angle of the Modified Polyvinyl Chloride Substrates by the One-Layer Approach

In contrast to the metallic titanium substrate, the PVC exhibited a different trend after being modified by various ZDS-containing solutions (Figure 5b). This can be attributed to the other reaction mechanisms between the metallic titanium and the plastic PVC substrate with the ZDS-containing solution. The compounds containing the catechol structure, such as ZDS and DA, would mainly attach/bind to the titanium by forming a bidentate structure [51]. On the other hand, the catechol-containing compound, for example, the DA that was most explored, can have various mechanistic steps, still full of debate, to deposit on the plastic substrates [52,53,54].

After immersing in the ZDS solution, the PVC substrate (ZDS-PVC) became more hydrophilic than the bare PVC (Figure 5b). This could be attributed to the hydrophilic zwitterionic terminal functionality. In contrast to the titanium substrate, adding the DA into the ZDS solution led to a lower contact angle, although this value still increased with the amount of DA added. This indicated that these ZDS + DA deposits on PVC may have quite different surface chemical configurations from those on titanium.

Immersing the modified PVC substrates into the NaIO_4_ oxidant solution showed quite different outcomes, depending on the solution composition used in the first step. For those modified with the ZDS + DA solutions in the first step, the additional NaIO_4_ oxidant immersion led to super hydrophilic surfaces (e.g., ZDS4DA(2-step)-PVC). By contrast, an additional NaIO_4_ immersion step led to a light hydrophobic surface if the first layer was prepared with the solution containing ZDS only (i.e., ZDS(2-step)-PVC vs. ZDS-PVC). This significantly differs from what is noted if the titanium substrate was used. Further, if the NaIO_4_ oxidant was added with ZDS in the first step, the ZDS(NaIO_4_)-PVC presented a similar surface contact angle as the one without the oxidant, ZDS-PVC. These surface hydrophilicity variations by adding the NaIO_4_ oxidant, either in the first or second step, are likely caused by the changes in surface chemical configurations, which will be explored by the XPS analysis in the subsequent section.

The addition of salt ions, 10 mM NaCl, was hypothesized to be able to change the interactions between the salt ions, the zwitterionic functionalities of ZDS, and the amine groups of DA in the coating/deposition solution. Nevertheless, the surface hydrophilicity remained similar between the surfaces modified with and without 10 mM NaCl (i.e., ZDS(2-step)-PVC vs. ZDSNaCl(2-step)-PVC; ZDS1DA(2-step)-PVC vs. ZDS1DANaCl(2-step)-PVC). Adding the 1 mg/mL polyethyleneimine (PEI, MW: 600) bearing primary and secondary amines, did not improve the surface hydrophilicity (ZDSPEINaCl-PVC and ZDS1DAPEINaCl-PVC). This may be due to the long hydrocarbon chains of PEI being exposed on the top layer of the deposit. Nevertheless, using a higher ionic strength of the depositing Tris buffer (50 mM vs. 10 mM), the contact angle was decreased (ZDSPEINaCl-PVC vs. ZDSPEINaCl50-PVC; and ZDS1DAPEINaCl-PVC vs. ZDS1DAPEINaCl50-PVC). These contact angle findings for adding the NaCl solution or using a higher ionic strength buffer suggest that proper tuning of the ionic interactions among the charged solutes and ionized functionalities is needed to optimize the surface characteristics, also noted in the previous SEM morphological analyses.

##### Surface Hydrophilicity of the Modified Polyvinyl Chloride Substrates by the Layer-by-Layer Approach

Previous studies have indicated that DA can assist the surface deposition/surface modification to render a surface with various functionalities [55,56]. Since there was a limited decrease in the surface contact angle after being immersed in the ZDS only solution (ZDS-PVC in Figure 5b), a layer-by-layer approach, in which different DA-containing solutions were utilized as the first layer deposit, was explored for the PVC surface modification.

**Figure 6:**
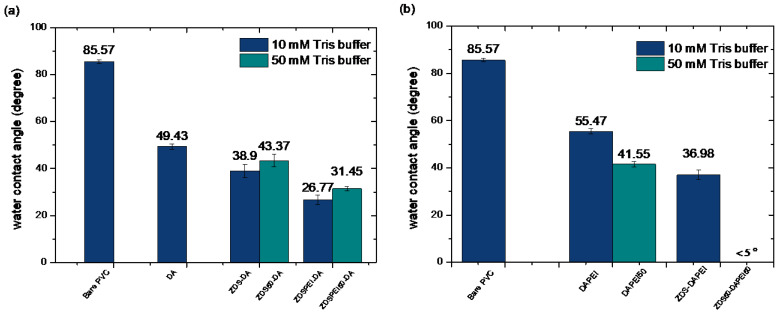
The static water contact angle value of PVC substrates modified by the layer-by-layer approach with the first layer of (**a**) DA or (**b**) DAPEI/DAPEI50.

Figure 6 shows the contact angle values of different layer-by-layer modified PVC substrates with the first layer of DA (Figure 6a) or DAPEI/DAPEI50 (Figure 6b). After being immersed in the DA solution, the PVC substrate (DA-PVC; 49.43 ± 1.16) exhibited a lower contact angle than the PVC modified by ZDS (ZDS-PVC; 68.40 ± 2.84, Figure 5b). This reflected the subtle difference in the surface structure (see XPS section), although both compounds carry the hydrophilic terminal functionalities. Further immersing the DA-PVC substrate into the ZDS or ZDSPEI solution in the 10 mM or 50 mM Tris buffer led to reduced contact angle values (Figure 6a) compared to the DA-PVC. Further, compared to the respective counterpart, the PEI addition resulted in a decreased contact angle, which could be attributed to the hydrophilic functionality of PEI. On the other hand, using a higher 50 mM Tris buffer led to a higher contact angle than the respective counterpart (Figure 6a).

In contrast to the findings noted in the second layer formation using the ZDS-containing solution, the PEI addition to the DA in the first immersion led to an increase in the contact angle (DAPEI-PVC; 55.47 ± 1.12 vs. DA-PVC; 49.43 ± 1.16; Figure 6a,b). Opposite to the findings in the second layer formation containing ZDS solution, the first layer with the DAPEI at a higher 50 mM Tris buffer showed a decrease in the contact angle compared to the one formed with the 10 mM Tris buffer. As the PVC substrate modified by the DAPEI solution in the 50 mM Tris buffer (DAPEI50-PVC) was further immersed in the ZDS solution in the 50 mM Tris buffer (ZDS50-DAPEI50-PVC, Figure 6b), the surface became very hydrophilic (<5°). These findings reflected the subtle interactions, yet to be determined, among the solutes, ions, and the PVC control or deposited first layer, which are crucial in governing the final layer surface hydrophilicity.

#### 3.2.3. XPS Analysis

The surface atomic percentages of various modified titanium and PVC substrates are shown in Table 4, Table 5 and Table 6. The quaternary N^+^% was determined by the N1s atomic percentage times the C-N^+^ area percentage value derived from the N1s curve fitting. The N1s peak was deconvoluted to the C-N-C (400.1 eV), C-N^+^ (402.4 eV), and C-NH_2_ (401.9 eV) for all samples except the modified Ti substrates, in which an additional peak, Ti-N (397 eV), was included for the N1S peak deconvolution. [57,58].

##### Surface Chemical Characteristics of the Modified Titanium Substrates

For the unmodified titanium (Bare Ti), likely due to the adsorbed adventitious hydrocarbon or the surface titanoxane (Ti-O-Ti) formed after being exposed to an oxygen-containing environment or irradiated with visible light [49], the C1s, N1s, and O1s peaks were noted in addition to the Ti2p peak (Table 4). On the Ti substrate modified only with ZDS (ZDS-Ti), an increase in the quaternary cationic N^+^% was noted while the Ti2p peak was still noted. This implies that the ZDS-deposited layer, likely due to the chelation between the titanium and the catechol structure in ZDS [50], is too thin for the XPS to detect the Ti2p photoelectrons ejected from the Ti substrate. Adding the NaIO_4_ oxidant in the second immersion step (ZDS(2-STEP)-Ti) in an attempt to convert the catechol to a quinone structure, as indicated in the earlier studies for enhancing the polydopamine deposition [42,43], did not change the ZDS-deposited layer formed in the first immersion (ZDS-Ti). This implied that the catechol groups of ZDS were almost consumed after chelating with the Ti. By contrast, the direct addition of the NaIO_4_ oxidant into the ZDS coating solution (i.e., ZDS(NaIO_4_)-Ti) led to similar XPS atomic percentage values as those on the Bare Ti control, suggesting no ZDS was formed. Nevertheless, the contact angle value for ZDS(NaIO_4_)-Ti (<5°) was similar to that for the Ti cleaned by the piranha solution (Ti (piranha)), not the hydrophobic Bare Ti control (Figure 5a). Such discrepancies may be caused by the experimental procedures employed. The contact angle measurement was performed immediately after the sample preparation. At the same time, the XPS analysis had to be performed in a vacuum chamber after the sample had been stored in a heating oven for a specific time to remove the water vapor. An extended sample’s exposure to the ambient environment leads to an increasing possibility of the adsorption of adventitious hydrocarbons. A further carefully designed experimental setup would be needed to elucidate the detailed surface chemical configuration on the ZDS(NaIO_4_)-Ti to exclude this possibility.

The Ti2p signal was not noted on the titanium substrates modified by adding DA to the ZDS coating solution (ZDS1DA-Ti, ZDS2DA-Ti, and ZDS4DA-Ti). This suggested that the addition of DA could significantly increase the deposit thickness to retard the Ti2p photoelectron from reaching the XPS photodetector. Although the N^+^% was decreased with the addition of DA compared to the one without, the N^+^% of these DA+ZDS-modified Ti substrates did not vary with the ratio of DA/ZDS in the coating solution. Further, the S2p/N^+^ values remained similar to and close to one, regardless of the amount of DA added to the ZDS coating solution. This implied that the surface deposit layer containing the sulfobetaine chemical configuration was associated with ZDS.

Further immersion of these ZDS + DA-modified Ti substrates in the NaIO_4_ oxidant solution led to a surface layer with reduced C1s% and increased O1s%. Still, the S2p/N^+^ remained similar to the non-oxidant modified substrate (i.e., ZDSxDA(2-STEP)-Ti vs. ZDSxDA-Ti). This suggested that the NaIO_4_ oxidant immersion would oxidize the pre-deposited ZDS + DA layer mainly on the hydrocarbon configuration while keeping the zwitterionic functionalities intact. For the substrates modified with the two-step NaIO_4_ immersion process, the addition of NaCl to the ZDS or ZDS1DA solution in the first deposition step did not change the chemical composition compared to those without, although the NaCl could change the ionic interactions among the ZDS molecules and the Ti substrate.

The C1s peak of the modified Ti substrates was further deconvoluted to various peaks with different chemical configurations (Table S4). The binding energy of the C1s in different chemical bonding states, namely C-C/C-H, C-O/C-N/C-S, C=O, and Ti-C, was adapted from the previous studies [59,60,61].

Titanium carbide (Ti-C) could be formed after the titanium metal was treated under certain conditions [62,63]. The Ti-C peak was noted on the surfaces upon which the Ti atoms were reported (Table 4), indicating these deposits were thin, as indicated in the previous discussion, which led to the observation of Ti-C from the substrate but at a lower area percentage than the Bare Ti. For the Ti substrates modified by the ZDS + DA solutions, the C-C/C-H area percentage increased with the DA added, while the C-N/C-O/C-S and C=O area percentages associated with the ZDS configuration were decreased. Adding the NaIO_4_ oxidant as a second-step treatment for the pre-deposited ZDS+DA titanium substrates resulted in a higher C=O but lower C-C/C-H area percentages than its counterpart. This further strengthens the discussion above that the NaIO_4_ oxidant immersion would oxidize the pre-deposits mainly on the hydrocarbon configuration while keeping the zwitterionic functionalities intact. Although the surface atomic percentage values (Table 4) revealed similar values for those surfaces deposited with or without the NaCl addition, the C1s curve fitting results suggested the surface chemical configurations were different (Table S4)

##### Surface Chemical Characteristics of the Modified Polyvinyl Chloride Substrates by the One-Layer Approach

The surface of the unmodified PVC (Bare PVC) will, theoretically, only have carbon and chlorine signals. However, oxygen signals were noted due to surface oxidation or organic pollutants adhering to its surface. A small amount of sulfur was also noted on the bare PVC, likely due to the use of vulcanizing agents or other sulfur-containing additives in the production process to change the PVC characteristics or to improve its performance (Table 5). The quaternary cationic ammonium (N^+^) appeared, but not many, after being immersed in the ZDS solution (ZDS-PVC). In the meantime, the Cl2p signals were still noted on the ZDS-PVC, indicating the thickness of this deposit was thin, as the case noted for titanium was used for the substrate. The use of the NaIO_4_ oxidant, either as a second-step treatment for the ZDS-deposited PVC or directly mixed with ZDS for the surface treatment, did not significantly reduce the Cl2p atomic percentage. Nevertheless, the S2p and N^+^ atomic percentages of these two modified PVC substrates (ZDS(2-STEP)-PVC and ZDS(NaIO_4_)-PVC) were higher than those of the no-oxidant added ZDS modified PVC (ZDS-PVC). Nonetheless, the S2p/N^+^ values were similar and close to one among these modified PVC substrates. This indicated that a zwitterionic sulfobetaine configuration was formed after being modified with the ZDS immersion, and the amount of this zwitterionic functionality was higher with the use of the NaIO_4_ oxidant.

Adding the DA to the ZDS immersion solution could significantly reduce the Cl2p signals on the modified PVC substrates (ZDSxDA-PVC). Nonetheless, the Cl2p signals are still noted, implicating the deposit is not thick enough to retard the Cl2p photoelectrons from reaching the XPS photodetector. Further, the quaternary ammonium N^+^% was increased while the S2p/N^+^ was similar to that modified by ZDS only. However, there were no significant differences in the N^+^% and S2p/N^+^ values among these three ZDS + DA modified PVC substrates. This suggested that adding DA could lead to more ZDS deposited onto the PVC substrates, in contrast to what was observed on the titanium substrate (Table 4). This further substantiates the roles of surface characteristics of the substrate in governing the deposition process and the final surface properties of the deposits.

In contrast to the ZDS(2-STEP)-PVC, the addition of the NaIO_4_ oxidant as the second-step treatment for the pre-deposited ZDSxDA layer does not vary the surface atomic percentages significantly (i.e., ZDSxDA(2-STEP)-PVC vs. ZDSxDA-PVC). Adding the NaCl to the ZDS or ZDS1DA solution in the first deposition step did not significantly vary the surface zwitterionic functionality (i.e., S2p/N^+^) compared to those without (Table 5). The further addition of PEI and/or a higher 50 mM Tris buffer in the ZDS + NaCl or ZDS1DA + NaCl first coating solution did not significantly change the surface zwitterionic functionality. However, adding PEI and/or using a 50 mM Tris buffer could result in different surface hydrophilicity (Figure 5b). Further, the deposit would be too thin for all the modified PVC studied here to have a reasonably high Cl2p atomic percentage if DA was not added to the coating solution.

The C1s peak of the modified PVC substrates was deconvoluted to C-C/C-H, C-N/C-O/C-S/C-Cl, and C=O peaks (Table S5). For Bare PVC, the notification of C=O was likely due to the surface oxidation or the adsorbed adventitious organic compounds (Table S5). As shown in Table 5, the deposit thickness of the PVC substrates prepared without DA added is thin. Henceforth, the C1s photoelectrons from the PVC substrate would make deciphering the carbon binding state of the surface deposit layer very difficult. By contrast, adding DA in the surface modification solution led to a thick layer, significantly reducing the chance of C1s from the PVC substrates reaching the XPS photodetector. Henceforth, the curve-fitting results for these DA-added modified PVC substrates are more reflective of the chemical binding states of the surface layer.

The increased C=O and decreased C-C/C-H area percentages on the ZDSnDA-modified PVC substrates (Table S5) imply that more zwitterionic chemical configuration is noted as compared to the one modified by ZDS only, also shown in surface atomic percentage analysis in Table 5. Adding the NaIO_4_ oxidant in the two-step treatment for the deposited ZDSnDA-modified PVC substrates led to a further increase in C=O area percentage but a decrease in C-C/C-H area percentage (Table S5). This is likely due to the oxidation of the hydrocarbon backbone of the deposited ZDSnDA on PVC, similar to that found if the titanium substrate was used (Table S4). Adding the PEI to the ZDS1DA + NaCl coating solution led to a surface with a further increase in C-N/C-O/C-S/C-Cl while a decrease in C-C/C-H area percentages. This could be due to the PEI being deposited on the surface layer. Regarding the likely roles of using a Tris buffer at two different ionic strengths, the C1s curve fitting results indicated that these two surfaces, ZDS1DAPEINaCl-PVC and ZDS1DAPEINaCl50-PVC, exhibited different chemical binding configurations. However, the surface atomic percentage values were similar (Table 5). The detailed causes remain elusive, and further studies are warranted.

##### Surface Chemical Characteristics of the Modified Polyvinyl Chloride Substrates by the Layer-by-Layer Approach

As indicated in the XPS analysis shown in Table 5, the deposition solution containing ZDS alone (ZDS-PVC) cannot lead to a thick deposit with a fair amount of zwitterionic sulfobetaine functionality. On the other hand, adding the DA to the ZDS-containing deposition solution can increase deposit thickness and the amount of zwitterionic functionality. Further, studies have shown that a pre-deposited DA layer can assist the subsequent deposition of different compounds without using any chemical to activate the covalent bond formation [55,56]. Henceforth, a layer-by-layer approach was explored to modify the non-reactive PVC substrate, in which the PVC substrate was first immersed in the DA solution, then the ZDS solution.

For the three PVC substrates modified by the first step of DA-containing solutions, namely DA-PVC, DAPEI-PVC, and DAPEI50-PVC, the Cl2p signals were still noted, but much less than the Bare PVC and ZDS-PVC (Table 5 and Table 6). This indicated that the thickness of these DA-containing first layers was greater than that of the ZDS deposit layer. With the addition of PEI to the DA immersion solution (i.e., DAPEI and DAPEI50 solution), the N1s atomic percentage of the deposit was significantly increased.

Following the deionized water rinsing of the first layer deposit, various ZDS-containing solutions were used as the second-layer modification step. The Cl2p signals were still noted, and the Cl2p atomic percentage values were close to those of the PVC modified only with the first layer (Table 6). This further highlighted that ZDS would not form a thick deposit, but DA would, on the non-reactive plastic substrate, such as the PVC studied here. Nevertheless, the notification of N^+^, S2p, and the value of S2p/N^+^ close to one indicated that zwitterionic sulfobetaine functionalities were formed on these layer-by-layer modified PVCs.

As for Table S5 of the PVC modified by the one-layer approach, the C1s of the PVC substrates modified by the layer-by-layer approach was deconvoluted to the C-C/C-H, C-N/C-O/C-S/C-Cl, and C=O peaks (Table S6). Compared to the Bare PVC, the C=O area percentage was increased on the first-layer DA-containing deposits, DA-PVC, DAPEI-PVC, and DAPEI50-PVC. This is speculated due to the oxidation of the catechol structure of DA to the quinone structure [64]. Adding the PEI to the DA coating solution rendered the first layer deposit with decreased C-C/C-H, but increased C=O area percentages. The detailed mechanism was unclear, but it may be related to PEI’s enhanced catechol–quinone structure conversion.

The C=O area percentage of the two layer-by-layer modified PVC substrates on which DA deposition was used as the first layer, namely ZDS-DA-PVC and ZDS50-DA-PVC, was higher than that of DA-PVC. This finding can be due to the incorporation of the zwitterionic sulfobetaine structure of ZDS. In conjunction with the atomic percentage analysis shown in Table 6, ZDS was successfully deposited on these layer-by-layer modified PVC substrates.

### 3.3. Antibacterial Assay

The antibacterial activity of the different modified titanium or PVC substrates was examined against gram-positive *S. aureus* (ATCC 21351), as shown in Table 7, Table 8 and Table 9, determined by the bacterial reduction percentage.

#### 3.3.1. Antibacterial Assay for the Modified Titanium Substrates

Due to the similar XPS atomic percentages among the ZDSxDA-Ti series, only ZDS1DA-Ti was tested against *S. aureus*. For similar reasons, the ZDS(2-STEP)-Ti and ZDS1DA(2-STEP)-Ti were selected for the antibacterial assay.

The addition of dopamine (DA) to the ZDS coating solution led to a surface with less antibacterial activity (ZDS1DA-Ti vs. ZDS-Ti) (Table 7). A similar finding was noted on the two-step NaIO_4_ oxidant-modified Ti surfaces (ZDS1DA(2-STEP)-Ti vs. ZDS(2-STEP)-Ti). This is likely attributed to the decreased S2p and N^+^ atomic percentages (Table 4), implicating a decrease in the zwitterionic sulfobetaine density on the surfaces modified with the DA addition. The reduced surface zwitterionic functionalities would lead to a less perfect adsorbed hydration layer, resulting in more microbes adsorbed/attached to the modified layer.

On the other hand, the role of the second-step NaIO_4_ immersion treatment in antibacterial effectiveness was not clear—a statistically significant increase in bacterial reduction after the ZDS-Ti was further immersed in the NaIO_4_ oxidant solution. A statistically similar antibacterial capability was noted for the ZDS1DA-Ti immersed in the NaIO_4_ solution. This may be due to the DA adsorbed on the modified surface. Further studies are warranted to clarify the causes.

Adding the NaCl to the first immersion solution drastically improved the antibacterial capability of the Ti substrates modified with the second-step NaIO_4_ immersion process (ZDSNaCl(2-STEP)-Ti vs. ZDS(2-STEP)-Ti; ZDS1DANaCl(2-STEP)-Ti vs. ZDS1DA(2-STEP)-Ti), although similar XPS atomic percentages were noted. The addition of NaCl could alter the intra-chain and/or inter-chain ionic interactions of the charged functionalities, such as the zwitterionic sulfobetaine studied here, resulting in a more extended configuration [65], as a result, enhancing the uniformity of the adsorbed hydration layer, and less bacterial adhesion.

#### 3.3.2. Antibacterial Assay for the One-Layer Modified Polyvinyl Chloride Substrates

Similar to the antibacterial studies on the modified titanium substrates (Section 3.3.1), ZDS1DA-PVC, ZDS(2-STEP)-PVC, and ZDS1DA(2-STEP)-PVC were selected for the antibacterial assay against *S. aureus*. Furthermore, due to the lowest S2p and N^+^ atomic percentages exhibited on the ZDS-PVC substrate among the samples studied (Table 5), the antibacterial assay was not tested on the ZDS-PVC.

Similar to the study on the modified titanium substrates, the addition of NaCl to the ZDS-containing immersion solution significantly improved the antibacterial activity of the modified PVC counterparts (i.e., ZDSNaCl(2-STEP)-PVC vs. ZDS(2-STEP)-PVC; ZDS1DANaCl(2-STEP)-PVC vs. ZDS1DA(2-STEP)-PVC) (Table 8).

Further adding the PEI to the coating solution did not improve the antibacterial activity significantly, although the free amine-terminated groups associated with PEI could be cationized (Table 8). This could be due to the counter effects caused by the hydrophobic alkyl chains of PEI. On the other hand, using a Tris buffer at a higher molarity, 50 mM vs. 10 mM, could improve the surface antibacterial activity (i.e., ZDSPEINaCl50-PVC vs. ZDSPEINaCl-PVC; ZDS1DAPEINaCl50-PVC vs. ZDS1DAPEINaCl-PVC). The highest antibacterial capability was noted on the ZDS1DAPEINaCl50-PVC. This underlies the essential roles of ionic interactions among the salts in the buffer, NaCl, PEI, and ZDS, in affecting the deposit surface properties and antibacterial capability.

#### 3.3.3. Antibacterial Assay for the Layer-by-Layer Modified Polyvinyl Chloride Substrates

For the layer-by-layer modified PVC substrates, those modified with the first layer of dopamine-containing solutions, namely DA-PVC, DAPEI-PVC, and DAPEI50-PVC, did not exhibit any antibacterial effect against *S. aureus*, instead showing a microbial growth enhancement effect, even when PEI was added. This may be due to the surface adhered alkyl hydrocarbon chains leading to enhanced microbial attachment and growth (Table 9).

With the subsequent immersion of these first-layer modified PVC substrates into different ZDS-containing solutions, the six studied layer-by-layer modified PVC substrates exhibited antibacterial effects. This finding confirmed that ZDS, containing the zwitterionic sulfobetaine terminal functionality, could lead to the antibacterial attachment effect, likely due to the adhered hydration layer in the zwitterionic polymers [19,20,65,66], even without the inclusion of PEI in the first or second immersion solution (i.e., ZDS-DA-PVC and ZDS50-DA-PVC vs. DA-PVC; ZDS-DAPEI-PVC vs. DAPEI-PVC; ZDS50-DAPEI50-PVC vs. DAPEI50-PVC). Nevertheless, the addition of PEI into the ZDS immersion solution would increase the antibacterial effect (i.e., ZDSPEI-DA-PVC vs. ZDS-DA-PVC, ZDSPEI50-DA-PVC vs. ZDS50-DA-PVC), likely due to the cationic amine functionalities of PEI.

### 3.4. Cytotoxicity Assay

The three modified Ti and PVC substrates, ZDSNaCl(2-STEP)-Ti, ZDS1DANaCl(2-STEP)-Ti, and ZDS1DAPEINaCl50-PVC, that exhibited high antibacterial activities, and the Bare Ti and Bare PVC samples were selected for testing their cytotoxicity. A cytotoxicity assessment following the ISO 10993-5 and ISO 10993-12 protocols was performed, and the sample which showed greater than 70% cell viability was considered non-cytotoxic [67,68].

**Figure 7:**
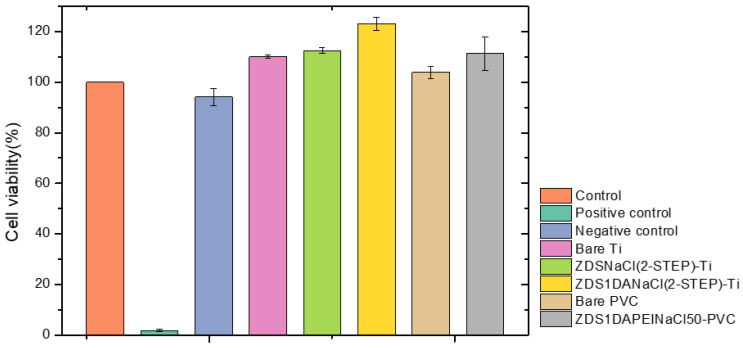
The cytotoxicity assay of the different samples (*n* = 3) (Control: culture medium; Positive control: latex glove; Negative control: PE plastic wrap).

The cells exhibited a spindle-like morphology similar to the control for the five Ti and PVC samples and the negative control. By contrast, for the positive control, the cells showed a spherical shape morphology and were suspended in the extract, which meant that the cells were apoptotic (Figure S1). Further, the cell viability results (Table S7 and Figure 7) indicated that all five Ti and PVC samples had a statistically higher cell viability than the control and were greater than 70%, a limit value defined as non-cytotoxic to the L929 cells. Further, the ZDS1DANaCl(2-STEP)-Ti showed the statistically highest cell viability.

By combining the cell morphology (Figure S1) and the cell viability results (Table S7 and Figure 7), all five modified Ti and PVC substrates exhibited non-cytotoxicity and good biocompatibility against the L929 cells, commonly used as the model cells for a cytotoxicity assay.

## 4. Conclusions

A unique catechol-terminated zwitterionic sulfobetaine, ZDS, was successfully synthesized and used for the surface modification of titanium (Ti) and polyvinyl chloride (PVC), substrates bearing distinct surface chemical configurations from each other. Different surface modification schemes using immersion processes were explored to change these two substrates’ surface characteristics and antibacterial properties.

A thick deposit cannot be formed on Ti and PVC substrates when the immersion/deposition solution contains ZDS, likely due to the short chain length and difficulty in self-polymerization/self-aggregation of ZDS. By contrast, a thick layer was formed when dopamine (DA) was added to the ZDS solution or used alone for immersion. Adding the NaIO_4_ oxidant to the co-deposition solution or as the second modification solution to those first modified with the ZDS/DA containing solution could lead to different surface characteristics, depending on the substrates and solution formulation. Further, since ZDS contains zwitterionic sulfobetaine functional groups, adding NaCl, PEI, and a Tris buffer at higher molarity to the immersion solution could change the ionic interactions among the compounds in the immersion solution and the substrate, especially the metallic Ti substrate, leading to deposits with different surface characteristics.

Based on the findings that the DA immersion could lead to a thick layer on PVC, but ZDS could not, a layer-by-layer approach was explored, in which the PVC substrate was first immersed in the solutions with DA and then in the ZDS solutions. The first DA-containing layer acted as the adhesion intermediate to promote the ZDS deposition, followed by the ZDS immersion. The surface characterization results indicated that the ZDS-containing layer was formed on the PVC after the layer-by-layer process.

Antimicrobial capability against *S. aureus* was assessed on various modified Ti and PVC substrates. Using ZDS or ZDS plus DA as the deposition solution could lead to modified substrates with antibacterial activity varying from 36 to 87% bacterial reduction, depending on the substrate and the solution formulation. By contrast, if DA was used in the immersion solution without ZDS, the modified PVC substrates enhanced microbial adhesion and proliferation. This underscores the critical role of ZDS in reducing *S. aureus* attachment and proliferation. The highest antibacterial capability was noted on the ZDSNaCl(2-STEP)-Ti and ZDS1DAPEINaCl50-PVC for the Ti and PVC substrates, respectively; these two modified surfaces were non-cytotoxic. This finding further stressed that selecting proper processing conditions, such as the immersion solution formulation and the additional oxidation modification shown here, would be important to significantly improve the antibacterial capability of ZDS-modified substrates without causing cytotoxicity. Nevertheless, further experiments are needed, and are currently being undertaken to explore the detailed mechanisms that lead to the enhanced antibacterial activity and distinct surface characteristics using ZDS and the additives utilized here.

## Figures and Tables

**Table 1:** Nomenclature for different surface-modified titanium substrates.

Bare Ti	Piranha-Treated Ti
ZDSxDA-Ti	Ti was modified with a coating solution of ZDS and DA at a weight ratio of ZDS/DA = 2:x for 24 h.
ZDS(NaIO_4_)-Ti	Ti was modified with a coating solution of ZDS and 20 mM NaIO_4_ for 2 h.
ZDSxDA(2-step)-Ti	The ZDSxDA-Ti sample was immersed in 50 mM of the NaIO_4_ solution for 0.5 h.
ZDSxDANaCl(2-step)-Ti	Ti was first modified with a coating solution containing ZDS and DA at a weight ratio of ZDS/DA = 2:x and 10 mM NaCl for 24 h. The modified sample was immersed in 50 mM of the NaIO_4_ solution for 0.5 h.

**Table 2:** Nomenclature for different surface-modified PVC substrates by the one-layer approach.

Bare PVC	PVC Underwent Washing/Cleaning Steps Only
ZDSxDA-PVC	PVC was modified with a coating solution of ZDS and DA at a weight ratio of ZDS/DA = 2:x for 24 h.
ZDS(NaIO_4_)-PVC	PVC was modified with a coating solution of ZDS and 20 mM NaIO_4_ for 2 h.
ZDSxDA(2-step)-PVC	The ZDSxDA-PVC sample was immersed in 50 mM of the NaIO_4_ solution for 0.5 h.
ZDSxDANaCl(2-step)-PVC	PVC was first modified with a coating solution containing ZDS and DA at a weight ratio of ZDS/DA = 2:x and 10 mM NaCl for 24 h. The modified sample was immersed in 50 mM of the NaIO_4_ solution for 0.5 h.
ZDSxDAPEINaCl-PVC	PVC was modified with a coating solution containing ZDS and DA at a weight ratio of ZDS/DA = 2:x, 1 mg/mL PEI, and 10 mM NaCl for 24 h.
ZDSxDAPEINaCl50-PVC	PVC was modified with a coating solution containing ZDS and DA at a weight ratio of ZDS/DA = 2:x, 1 mg/mL PEI, and 10 mM NaCl for 24 h. The Tris buffer was adjusted to 50 mM instead of 10 mM as the other coating solutions.

**Table 3:** Nomenclature for different layer-by-layer modified PVC substrates.

	Bare PVC	PVC Underwent Washing/Cleaning Steps Only
First layer	DA-PVC	PVC was modified with a 2 mg/mL of the DA solution for 24 h
	DAPEI-PVC	PVC was modified with a coating solution containing 2 mg/mL DA and 1 mg/mL PEI for 24 h.
	DAPEI50^@^-PVC	The PVC was modified similarly to the DAPEI-PVC, except the Tris buffer was adjusted to 50 mM instead of 10 mM.
Second layer	ZDS-DA-PVC	The DA-PVC was further immersed in 5 mg/mL of the ZDS solution for 24 h.
	ZDS50^@^-DA-PVC	The sample was modified similarly to the ZDS-DA-PVC, except the Tris buffer was adjusted to 50 mM instead of 10 mM.
	ZDSPEI-DA-PVC	The DA-PVC was further immersed in a coating solution containing 5 mg/mL ZDS and 2.5 mg/mL PEI for 24 h.
	ZDSPEI50^@^-DA-PVC	The sample was modified similarly to the ZDSPEI-DA-PVC, except the Tris buffer was adjusted to 50 mM instead of 10 mM.
	ZDS-DAPEI-PVC	The DAPEI-PVC was immersed in 5 mg/mL of the ZDS solution for 24 h.
	ZDS50^@^-DAPEI50^@^ -PVC	The DAPEI50-PVC was immersed in 5 mg/mL of the ZDS solution with 50 mM of the Tris buffer for 24 h.

^@^: The molarity of the Tris buffer used was 50 mM, higher than the Tris buffer, 10 mM, used for the rest of the coating solution studied.

**Table 4:** Surface atomic percentage of different modified titanium substrates.

Sample	C1s	N1s	O1s	S2p	Ti2p	N^+^
Bare Ti	36.2%	3.7%	41.9%	0.0%	18.1%	0.0%
ZDS-Ti	38.5%	2.6%	41.0%	1.1%	16.8%	1.0%
ZDS1DA-Ti	71.5%	8.0%	20.1%	0.5%	0.0%	0.6%
ZDS2DA-Ti	74.5%	8.0%	17.0%	0.5%	0.0%	0.6%
ZDS4DA-Ti	73.7%	7.5%	18.4%	0.4%	0.0%	0.5%
ZDS(2-STEP)-Ti	37.9%	2.8%	41.7%	0.9%	16.7%	1.0%
ZDS(NaIO4)-Ti	36.2%	2.0%	43.7%	0.0%	18.1%	0.0%
ZDS1DA(2-STEP)-Ti	69.0%	7.8%	22.3%	0.8%	0.0%	0.7%
ZDS2DA(2-STEP)-Ti	68.5%	7.7%	23.1%	0.6%	0.0%	0.6%
ZDS4DA(2-STEP)-Ti	68.0%	8.0%	23.4%	0.6%	0.0%	0.7%
ZDSNaCl(2-STEP)-Ti	38.5%	2.3%	41.5%	0.8%	16.9%	0.9%
ZDS1DANaCl(2-STEP)-Ti	68.5%	7.4%	23.5%	0.7%	0.0%	0.6%

**Table 5:** Surface atomic percentage of various PVC substrates modified by the one-layer approach.

Sample	C1s	N1s	O1s	S2p	Cl2p	N^+^
Bare PVC	70.2%	0.0%	4.3%	0.5%	25.1%	0.0%
ZDS-PVC	69.2%	1.7%	10.7%	0.4%	17.9%	0.3%
ZDS1DA-PVC	68.1%	7.2%	22.3%	0.8%	1.6%	0.9%
ZDS2DA-PVC	68.4%	8.1%	21.0%	0.9%	1.6%	0.9%
ZDS4DA-PVC	69.6%	8.2%	19.5%	0.8%	1.9%	0.8%
ZDS(2-STEP)-PVC	68.0%	2.5%	13.5%	0.9%	15.1%	0.8%
ZDS(NaIO4)-PVC	69.3%	2.2%	9.8%	0.7%	18.1%	0.7%
ZDS1DA(2-STEP)-PVC	68.2%	7.5%	22.0%	0.9%	1.5%	0.8%
ZDS2DA(2-STEP)-PVC	67.3%	8.4%	22.2%	0.9%	1.3%	1.0%
ZDS4DA(2-STEP)-PVC	67.0%	8.6%	22.6%	0.8%	1.0%	0.9%
ZDSNaCl(2-STEP)-PVC	68.1%	1.8%	11.3%	0.6%	18.3%	0.7%
ZDS1DANaCl(2-STEP)-PVC	67.9%	7.6%	22.2%	0.8%	1.5%	0.7%
ZDSPEINaCl-PVC	70.1%	3.2%	10.2%	0.7%	15.7%	0.7%
ZDS1DAPEINaCl-PVC	65.5%	15.2%	15.4%	0.6%	3.3%	0.7%
ZDSPEINaCl50-PVC	66.6%	4.0%	11.6%	0.8%	17.0%	0.7%
ZDS1DAPEINaCl50-PVC	65.7%	14.8%	15.7%	0.7%	3.1%	0.8%

**Table 6:** Surface atomic percentage of various PVC substrates modified by the layer-by-layer approach.

Sample	C1s	N1s	O1s	S2p	Cl2p	N^+^
Bare PVC	70.2%	0.0%	4.3%	0.5%	25.1%	0.0%
DA-PVC	69.8%	8.1%	20.4%	0.0%	1.7%	0.0%
DAPEI-PVC	66.1%	15.3%	14.4%	0.0%	4.2%	0.0%
DAPEI50-PVC	66.2%	15.3%	16.1%	0.0%	2.3%	0.0%
ZDS-DA-PVC	69.7%	7.5%	20.1%	0.6%	2.2%	0.7%
ZDS50-DA-PVC	67.3%	7.8%	22.2%	0.6%	2.1%	0.7%
ZDSPEI-DA-PVC	66.4%	13.2%	16.0%	0.5%	3.9%	0.7%
ZDSPEI50-DA-PVC	67.2%	12.2%	17.7%	0.6%	2.2%	0.7%
ZDS-DAPEI-PVC	65.8%	12.0%	17.3%	0.7%	4.3%	0.6%
ZDS50-DAPEI50-PVC	67.4%	11.6%	17.8%	0.5%	2.6%	0.5%

**Table 7:** The bacterial reduction percentage of different titanium samples against *S. aureus* (*n* = 3).

Sample	Bacterial Reduction (%)
Bare Ti	0
ZDS-Ti	54.7 ± 0.9
ZDS(2-STEP)-Ti	64.0 ± 7.4
ZDS1DA-Ti	49.4 ± 6.3
ZDS1DA(2-STEP)-Ti	52.0 ± 3.2
ZDSNaCl(2-STEP)-Ti	84.2 ± 4.9
ZDS1DANaCl(2-STEP)-Ti	73.5 ± 5.4

**Table 8:** The bacterial reduction percentage of different one-layer modified PVC samples against *S. aureus* (*n* = 3).

Sample	Bacterial Reduction (%)
Bare PVC	0
ZDS(2-step)-PVC	58.8 ± 1.5
ZDS1DA-PVC	40.3 ± 2.0
ZDS1DA(2-step)-PVC	36.6 ± 1.5
ZDSNaCl(2-STEP)-PVC	71.7 ± 2.1
ZDS1DANaCl(2-STEP)-PVC	57.0 ± 4.6
ZDSPEINaCl-PVC	66.4 ± 2.0
ZDSPEINaCl50-PVC	71.8 ± 2.0
ZDS1DAPEINaCl-PVC	71.2 ± 0.3
ZDS1DAPEINaCl50-PVC	81.7 ± 6.5

**Table 9:** The bacterial reduction percentage of different layer-by-layer modified PVC samples against *S. aureus* (*n* = 3).

Sample	Bacterial Reduction (%)
Bare PVC	0
DA-PVC	−24.0 ± 3.7
DAPEI-PVC	−15.8 ± 3.9
DAPEI50-PVC	−32.0 ± 5.5
ZDS-DA-PVC	63.8 ± 1.9
ZDS50-DA-PVC	52.8 ± 6.1
ZDSPEI-DA-PVC	71.0 ± 1.0
ZDSPEI50-DA-PVC	66.6 ± 1.5
ZDS-DAPEI-PVC	63.8 ± 1.0
ZDS50-DAPEI50-PVC	62.5 ± 1.3

## Data Availability

The original contributions presented in this study are included in the article/Supplementary Materials. Further inquiries can be directed to the corresponding author.

## Supplementary Materials

The following supporting information can be downloaded at: https://www.mdpi.com/article/10.3390/polym17152006/s1. Figure S1: The representative optical images (×10) of L929 cultured in different medium solutions, which were prepared by immersing the sterilized samples for 24 h (Control: culture medium; Positive control: latex glove; Negative control: PE plastic wrap). Table S1: Processing parameters for different modified titanium substrates. Table S2: Processing parameters for different modified PVC substrates by the one-layer approach. Table S3: Processing parameters for different layer-by-layer modified PVC substrates. Table S4: The C1s curve fitting results of different modified titanium substrates. Table S5: The C1s curve fitting results of different modified PVC substrates modified by the one-layer approach. Table S6: The C1s curve fitting results of different modified PVC substrates modified by the lay-by-layer approach. Table S7: The cell viability for the cytotoxicity assay of different samples (*n* = 3) (Control: culture medium; Positive control: latex glove; Negative control: PE plastic wrap).
