# Supplementary material for: Surface Modification of Medical-Grade Titanium and Polyvinyl Chloride with a Novel Catechol-Terminated Compound Containing Zwitterionic Sulfobetaine Functionality for Antibacterial Application
Source: Polymers (Basel). 2025 Jul 22;17(15):2006. doi: 10.3390/polym17152006 (PMC12349499; doi:10.3390/polym17152006)
Supplement: Supplementary file 1 [file polymers-17-02006-s001.zip › polymers-3761680-supplementary.pdf]

Surface modification of medical-grade titanium and polyvinyl chloride with a novel catechol-terminated compound containing zwitterionic sulfobetaine functionality for antibacterial application

Naichia Fan[1,2], Fang-Min Hsu[3], Chi-Hui Cheng[1,4], Jui-Che Lin[3,5*]

[1] Division of Nephrology, Department of Pediatrics, Chang Gung Memorial Hospital, Taoyuan, Taiwan

[2] Graduate Institute of Clinical Medical Sciences, Chang Gung University, Taoyuan, Taiwan

[3] Department of Chemical Engineering, National Cheng Kung University, Tainan, Taiwan

[4] Department of Pediatrics, College of Medicine, Chang Gung University, Taoyuan, Taiwan

[5] School of Dentistry, Institute of Oral Medicine, College of Medicine, National Cheng Kung University, Tainan, Taiwan

Corresponding Author

* Prof. Jui-Che Lin: Department of Chemical Engineering, National Cheng Kung University, Tainan, TAIWAN 70101, Phone: +886-6-275-7575 ext. 62665, Fax: +886-6-234-4996, Email: jclin@mail.ncku.edu.tw ORCID ID: https://orcid.org/0000-0003-4436-655X

Table S1 Processing parameters for different modified titanium substrates.

Sample	ZDS	DA	NaCl	$NaIO_4$	Immersion
ZDS-Ti	2 mg/mL	-	-	-	24 hr
ZDS1DA-Ti	2 mg/mL	1 mg/mL	-	-	24 hr
ZDS2DA-Ti	2 mg/mL	2 mg/mL	-	-	24 hr
ZDS4DA-Ti	2 mg/mL	4 mg/mL	-	-	24 hr
ZDS($NaIO_4$)-Ti	2 mg/mL	-	-	20 mM	2 hr
ZDS(2-step)-Ti	2 mg/mL	-	-	50 mM	24 hr + 0.5 hr
ZDS1DA(2-step)-Ti	2 mg/mL	1 mg/mL	-	50 mM	24 hr + 0.5 hr
ZDS2DA(2-step)-Ti	2 mg/mL	2 mg/mL	-	50 mM	24 hr + 0.5 hr
ZDS4DA(2-step)-Ti	2 mg/mL	4 mg/mL	-	50 mM	24 hr + 0.5 hr
ZDSNaCl(2-step)-Ti	2 mg/mL	-	10 mM	50 mM	24 hr + 0.5 hr
ZDS1DANaCl(2-step)-Ti	2 mg/mL	1 mg/mL	10 mM	50 mM	24 hr + 0.5 hr

Table S2 Processing parameters for different modified PVC substrates by the one-layer approach

Sample	ZDS	DA	NaCl	PEI	$NaIO_4$	Immersion
ZDS-PVC	2 mg/mL	-	-		-	24 hr
ZDS1DA-PVC	2 mg/mL	1 mg/mL	-		-	24 hr
ZDS2DA-PVC	2 mg/mL	2 mg/mL	-		-	24 hr
ZDS4DA-PVC	2 mg/mL	4 mg/mL	-		-	24 hr
ZDS($NaIO_4$)-PVC	2 mg/mL	-	-		20 mM	2 hr
ZDS(2-step)-PVC	2 mg/mL	-	-		50 mM	24 hr + 0.5 hr
ZDS1DA(2-step)-PVC	2 mg/mL	1 mg/mL	-		50 mM	24 hr + 0.5 hr
ZDS2DA(2-step)-PVC	2 mg/mL	2 mg/mL	-		50 mM	24 hr + 0.5 hr
ZDS4DA(2-step)-PVC	2 mg/mL	4 mg/mL	-		50 mM	24 hr + 0.5 hr
ZDSNaCl(2-step)-PVC	2 mg/mL	-	10 mM		50 mM	24 hr + 0.5 hr
ZDS1DANaCl(2-step)-PVC	2 mg/mL	1 mg/mL	10 mM		50 mM	24 hr + 0.5 hr

ZDSPEINaCl-PVC	2 mg/mL		10 mM	1 mg/mL	24 hr
ZDS1DAPEINaCl-PVC	2 mg/mL	1 mg/mL	10 mM	1 mg/mL	24 hr
ZDSPEINaCl50@-PVC	2 mg/mL		10 mM	1 mg/mL	24 hr
ZDS1DAPEINaCl50@-PVC	2 mg/mL	1 mg/mL	10 mM	1 mg/mL	24 hr

@: The molarity of the Tris buffer used was 50 mM, higher than the Tris buffer, 10 mM, used for the rest of the coating solution studied.

Table S3 Processing parameters for different layer-by-layer modified PVC substrates.

Sample	ZDS	DA	PEI	Immersion
DA-PVC	-	2 mg/mL	-	24 hr
DAPEI-PVC	-	2 mg/mL	1 mg/mL	24 hr
DAPEI50@-PVC	-	2 mg/mL	1 mg/mL	24 hr
ZDS-DA-PVC	5 mg/mL	-	-	24 hr
ZDS50@-DA-PVC	5 mg/mL	-	-	24 hr
ZDSPEI-DA-PVC	5 mg/mL	-	2.5 mg/mL	24 hr
ZDSPEI50@-DA-PVC	5 mg/mL	-	2.5 mg/mL	24 hr
ZDS-DAPEI-PVC	5 mg/mL	-	-	24 hr
ZDS50@-DAPEI50@-PVC	5 mg/mL	-	-	24 hr

@: The molarity of the Tris buffer used was 50 mM, higher than the Tris buffer, 10 mM, used for the rest of the coating solution studied.

Table S4 The C1s curve fitting results of different modified titanium substrates.

**Sample**	**C-C/C-H (285eV)**	**C-N/C-O/C-S (286.6eV)**	**C=O (288.6eV)**	**Ti-C (282 eV)**
Bare Ti	66.4%	10.4%	12.2%	11.0%
ZDS-Ti	68.8%	17.9%	7.5%	5.8%
ZDS1DA-Ti	53.7%	33.7%	12.6%	0.0%
ZDS2DA-Ti	58.7%	32.8%	8.5%	0.0%
ZDS4DA-Ti	73.7%	24.1%	2.2%	0.0%
ZDS(2-STEP)-Ti	68.6%	16.0%	10.1%	5.3%
ZDS(NaIO4)-Ti	72.4%	8.9%	13.2%	5.6%
ZDS1DA(2-STEP)-Ti	45.4%	38.3%	16.3%	0.0%
ZDS2DA(2-STEP)-Ti	52.7%	34.3%	13.0%	0.0%
ZDS4DA(2-STEP)-Ti	54.9%	34.0%	11.1%	0.0%

ZDSNaCl(2-STEP)-Ti	70.8%	17.8%	6.4%	5.1%
ZDS1DANaCl(2-STEP)-Ti	54.5%	29.8%	15.7%	0.0%

Table S5 The C1s curve fitting results of different modified PVC substrates modified by the one-layer approach.

**Sample**	**C-C/C-H (285eV)**	**C-N/C-O/C-S/C-Cl (286.6eV)**	**C=O (288.6eV)**
Bare PVC	63.7%	34.1%	2.2%
ZDS-PVC	66.2%	31.7%	2.1%
ZDS1DA-PVC	49.1%	46.4%	4.5%
ZDS2DA-PVC	50.3%	43.4%	6.2%
ZDS4DA-PVC	53.0%	41.8%	5.2%
ZDS(2-STEP)-PVC	68.7%	26.9%	4.4%
ZDS(NaIO4)-PVC	69.5%	27.2%	3.3%
ZDS1DA(2-STEP)-PVC	47.5%	40.1%	12.4%
ZDS2DA(2-STEP)-PVC	44.0%	43.7%	12.3%
ZDS4DA(2-STEP)-PVC	46.5%	43.1%	10.4%
ZDSNaCl(2-STEP)-PVC	67.7%	30.0%	2.3%
ZDS1DANaCl(2-STEP)-PVC	42.2%	46.7%	11.1%
ZDSPEINaCl-PVC	67.6%	28.4%	4.0%
ZDS1DAPEINaCl-PVC	21.4%	65.6%	13.0%
ZDSPEINaCl50-PVC	65.8%	28.9%	5.3%
ZDS1DAPEINaCl50-PVC	27.4%	63.2%	9.4%

Table S6 The C1s curve fitting results of different modified PVC substrates modified by the lay-by-layer approach.

**Sample**	**C-C/C-H (285eV)**	**C-N/C-O/C-S/C-Cl (286.6eV)**	**C=O (288.6eV)**
Bare PVC	63.7%	34.1%	2.2%
DA-PVC	49.3%	44.6%	6.1%
DAPEI-PVC	37.9%	49.2%	12.9%
DAPEI50-PVC	33.1%	53.5%	13.4%
ZDS-DA-PVC	46.7%	39.2%	14.2%
ZDS50-DA-PVC	48.9%	39.8%	11.3%
ZDSPEI-DA-PVC	41.8%	44.2%	14.0%
ZDSPEI50-DA-PVC	41.3%	48.1%	10.6%

ZDS-DAPEI-PVC	32.5%	55.9%	11.6%
ZDS50-DAPEI50-PVC	41.9%	47.4%	10.7%

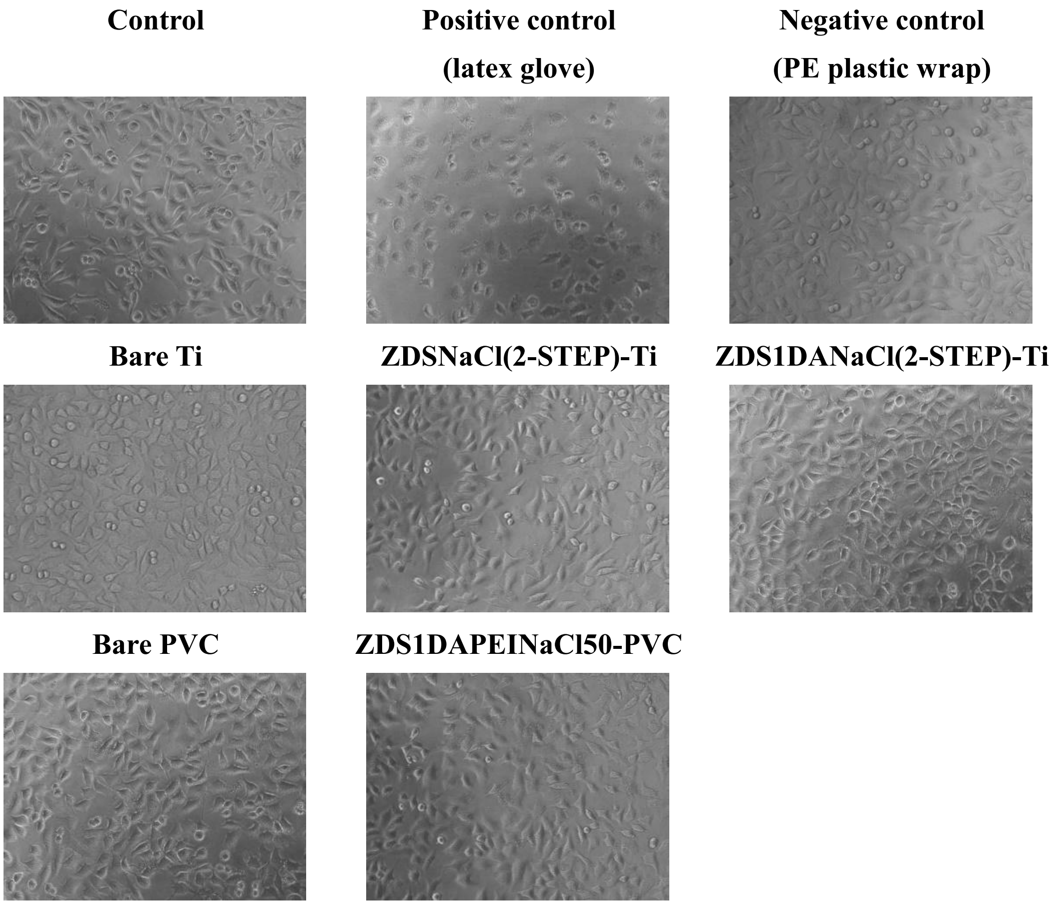

Figure S1. The representative optical images (×10) of L929 cultured in different medium solutions, which were prepared by immersing the sterilized samples for 24 hours (Control: culture medium; Positive control: latex glove; Negative control: PE plastic wrap).

Table S7 The cell viability for the cytotoxicity assay of different samples (n=3) (Control: culture medium; Positive control: latex glove; Negative control: PE plastic wrap).

**Sample**	**Cell Viability (%)**
Control	100
Positive control	1.74±0.41
Negative control	94.14±3.31

Bare Ti	110.09±0.64
ZDSNaCl(2-STEP)-Ti	112.56±1.19
ZDS1DANaCl(2-STEP)-Ti	123.20±2.52
Bare PVC	103.96±2.93
ZDS1DAPEINaCl50-PVC	111.32±6.54
